# Understanding interaction rituals: The impact of interaction ritual chains of the live broadcast on people’s wellbeing

**DOI:** 10.3389/fpsyg.2022.1041059

**Published:** 2022-10-27

**Authors:** Lu Meng, Yijun Zhao, Yushi Jiang, Yongyue Bie, Jingpeng Li

**Affiliations:** ^1^School of Economics and Management, Southwest Jiaotong University, Chengdu, China; ^2^Marketing Department, School of Business, Renmin University of China, Beijing, China

**Keywords:** live-broadcast, interaction ritual theory, meaning transfer model, wellbeing, COVID-19

## Abstract

With the global pandemic of COVID-19, it has been striking psychological burdens on individuals. Under this background, more and more people get wellbeing by watching live broadcasts. However, the psychological mechanism behind this phenomenon is still a black box. This study finds that when people watch a live broadcast and interact with anchors and other people, an interaction ritual chain is formed, and emotional energy is generated, thus making people experience and understand the meaning of the live interaction ritual chains. Under the effect of the meaning transfer model, people will generate wellbeing. Specifically, the basic meaning of live interaction (emotional meaning and functional meaning) drives people’s generation of wellbeing. The meanings of self-participation, self-display, self-concept, and self-renewal play a role in mediation in enhancing people’s wellbeing with the basic meaning of live broadcast interaction.

## Introduction

In 2020, with the global outbreak of the new crown, a critical factor in slowing the spread of the virus was “social isolation,” which has led to the closure of many parts of the world and has caused great anxiety and fear within people (Luo et al., 2021). In this context, many companies around the world have been forced to shut down their production and sales, causing a huge impact on the real economy. However, the live-streaming industry has seen tremendous growth rather than decline (Liu et al., 2022).

China had 587 million online live broadcast people in 2020, according to iiMedia Research (iiMedia, 2021). Live broadcast services developed by YouTube, Facebook, and Tik Tok are attracting more people (Hilvert-Bruce et al., 2018). Internet-based live-streaming offers content creators unprecedented access to their content, allowing them to deliver it to people (Diwanji et al., 2020).

The reason for this is that in the process of maintaining social distance from home isolation, people choose to watch live streams to satisfy social needs, relieve anxiety, and make contactless purchases and use them to gain wellbeing (De Wit et al., 2020).

During COVID-19, many people will have their favorite anchors and regular viewing habits after watching the webcast platform for a period of time (Peng et al., 2020). When they have no intention of shopping, some may still watch the channels of their favorite anchors on a regular basis, having used live streaming as a leisure and entertainment program and making them feel able to gain wellbeing by watching it. However, why people can generate wellbeing through watching live streams is a black box that existing research has yet to open. Furthermore, from the perspective of live streaming practitioners, with the diversity of live streaming platforms. Competition is fierce. Once people are dissatisfied with the service, they stop using it or switch to a different platform. Therefore, how to make people get wellbeing by watching their own live streams and carrying out willingness to develop a habit of continuous viewing is a central question for the long-term development of live e-commerce (Chen and Chang, 2019).

At the same time, existing research on the after-effects of live broadcasts mainly focuses on purchase intentions (Qing and Jin, 2022), gift rewards (Hilvert-Bruce et al., 2018), etc., while focusing on continued viewing behavior and research on its psychological mechanism are still relatively scarce. Few research is about interpreting the people to generate wellbeing from the perspective of the anchor’s identity (Hu et al., 2017). However, this research considers the factors of emotional arousal and the anchor’s cognitive identity. Unlike other media, the main feature of online live broadcasts is that people and anchors can interact in real-time in multiple directions, connect people and build a sense of community. Interaction is often driven by emotion, which produces a series of interactive rituals. Interactive rituals refer to the interaction rituals between people and others in the context of facing each other, and their behavior has symbolic meaning (Raj, 2012). Therefore, the live interactive ritual is a ritualized interactive behavior between a people and a specific anchor. It is a set of repetitive, meaningful, and non-functional behaviors (Rufi et al., 2016). In addition, during live broadcast, the people’s generation of wellbeing developed by people perhaps cannot be achieved by a single force, by the anchor, for example, but rather by the collective arousal behavior achieved through high-frequency multi-directional interactions involving the anchor, people, and another factor as a whole together. In each interactive ceremony of the webcast, the participants gather through the focus of mutual attention and gain a sense of group unity. The extension of the symbolic meaning encourages participants to join the motivation for the following interactive ritual. The long-term commitment of a group is fulfilled and maintained through a collective chain of interaction rituals, which can foster an intense and shared excitement (Collins, 2014; Durkheim, 2016), further leading people to generate wellbeing and would like to keep watching live. Through extensive qualitative analyses of literature and a series of in-depth interviews, this study explored and established the formation path of live interactive ritual chains for live broadcasts in three steps. Firstly, it can be understood based on the theory of interactive ritual chain people enter the live-broadcast channels to watch and interact with the anchor and other audiences, forming a live interactive ritual chain and generating and experiencing emotional energies; second, to generate wellbeing has been driven by experiencing emotional energies and understanding the meaning (emotional meaning and functional meaning) of live interactive ritual chains, in particular, enhanced by the mediation of the meanings of self-participation, self-display, self-concept, and self-renewal of interactive ritual chains.

The main contributions of this study include, firstly, identifying the uniqueness of live broadcasts from the perspective of the theory of interaction ritual chains, introducing a framework based on interaction ritual chains and meaning transfer model to understand people’s generation of wellbeing and participating activities in live broadcast e-commerce; secondly, this study, in addition to applied and expend the theory of interaction ritual chains to live-broadcasts, also applied the meaning transfer model to this new domain for improved understanding. Previous research on the meaning transfer model has considered symbols to be non-transitive flows (Mead and Williams, 2022); this study has revealed that in live broadcasts, the interactions can be multiple ways flow among symbols. Thirdly, this study focuses on people’s wellbeing generation, which will supplement the existing literature on live broadcasts.

## Theoretical background or literature review

### Live broadcast

Live-broadcast e-commerce is a relatively new phenomenon. Thus, it is necessary to understand people’s viewing procedures (Xue et al., 2020). People need to register and log in to a live broadcast channel to view live broadcasts. If a person is interested in a product promoted by the anchor, the person may browse the product’s website, search for detailed information, and purchase the product (Croes and Bartels, 2021). While watching the live broadcasts, viewers may engage with the anchor and other audiences in discussions or exchange information through screen bullets; those exchanges can be made before and after browsing product web pages or purchases. After each show, viewers may leave the live-broadcast channel or watch the next show.

Compared with traditional media, the main difference lies in the possibility of interacting with the anchor and other audiences to participate in live broadcast activities in real-time. Real-time engagement can fulfill audiences’ psychological needs for socialization and entertainment, which induce behaviors such as recognition, attention, sharing, and purchase (Hilvert-Bruce et al., 2018).

### Interaction ritual chain theory

The word “ritual” is derived from the Latin word “rituals,” typically understood to mean a ceremony or custom, which refers to actions expressing value and meaning and having repetitive patterns and regularities (Rook, 1985). The Interaction ritual chains (IRC) theory of Collins points out that a ritual is a programmatic behavior that includes a variety of symbols to express meaning. It is a type of activity of participants with a common focus and shared emotions. The ritual’s core mechanisms are the rhythmic and harmonious interaction process, mutual attention, and emotional energy. Collins (2014) examines static interaction rituals dynamic from the perspective of “chains” and proposes the interaction ritual chains theory – participants gather in a bounded space to jointly participate in a symbolic activity driven by emotions and generate emotional energies of collective identity. They can further strengthen future participants’ willingness to interact with each other. The focus of mutual attention and mutual emotional connection among various elements of interaction ritual chains are at the core position of the interaction ritual. In this process, the spiritual resonance of the participants needs to be stimulated through a high degree of coordination and consistency of actions. This kind of physical and mental cooperation forms a sense of identity for the members associated with the symbolic focus of mutual attention. The participants also harvest the matching emotional energy (Collins, 2014).

Therefore, the interaction ritual chains theory can be seen as a set of processes with causal connections and feedback loops. Interaction is often driven by emotions, which produce a series of rituals. With the three elements of group gathering, rejection of outliers, and unity of focus as the starting condition, the shared emotions and capital investment as the driving force, in the process of joint actions of participants adopting rituals, rhythmic feedback and further reinforces the collective excitement. Related research on the interaction ritual chains theories initially emerged in sociology, mainly discussing the formation mechanism of a series of social relationships for religions, and online social communities (Maloney, 2013). These research results confirm that most social relationships are formed and maintained through various rituals. Subsequently, interaction ritual chain theory was applied to marketing research (Brown, 2011) and Hill et al. (2022) take the Premier League football stadium as the subject of their study, and this article uses sociological interaction ritual theory to conceptualize “social atmospheres”: rapidly changing qualities of place created when a shared focus aligns consumers’ emotions and behavior, resulting in lively expressions of collective effervescence.

Similarly, the live-broadcast channels also offer a place for people to realize interpersonal communication. Especially live-broadcast channels meet people’s various needs, where the Especially live-broadcast channels meet people’s various needs where the audience’s emotions have also been released, exchanged, or accumulated, meeting the basic requirements of the interactive ritual chain.

This study argues that interactive ritual chain theory provides a promising way to understand the emotional energy and associated meanings formed during the process of consumers’ watching live broadcasts.

### Meaning transfer model

Meaning the transfer model influences skills, knowledge, or attitudes acquired in one situation on their formation in another situation (Schimmelpfennig and Hunt, 2020). The meaning transfer model was introduced to interpret the transfer of meanings in culture and society through interactions between community members. These meanings have been transferred from the culture and society to consumers’ goods or brands. The exact meanings were then accepted or passed on through rituals such as uses and consumption. For example, the process of live broadcast anchor advertising’s influence on the audience is also the process of celebrities’ image migration (Schimmelpfennig and Hunt, 2020); when they choose a brand to promote, their assigned meaning or value of them will be transferred to the brand they endorse and establish the same characteristics or meaning for the brand, consumers buy the products for the reasons of having the same image or characteristics as the celebrities in the advertisement. The characteristics or purpose will be transferred to the consumers (Shavitt et al., 1994).

This study uses the meaning transfer model theory to analyze live-broadcast scenarios. As mentioned before, according to the theory of interactive ritual chain, it is found that when people enter the live broadcast channel to watch the anchor’s live broadcasts and participate in activities, they gather their support for the anchor through interaction and communication that they will generate emotional energy. Subsequently, the activities and live interactions generate special meaning for them.

**FIGURE 1:**
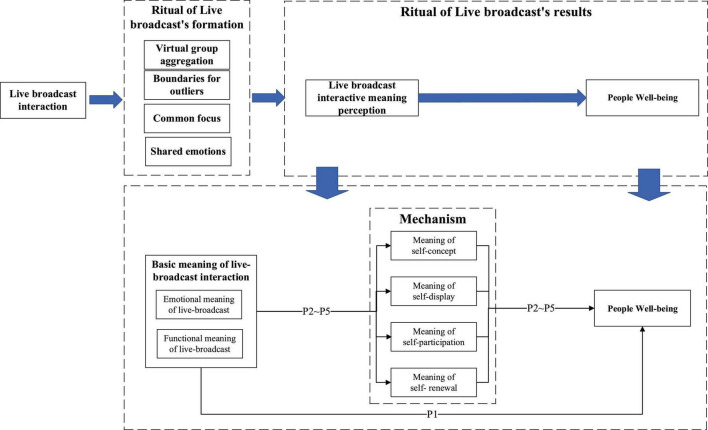
The framework.

According to the meaning transfer model, this process allows people to form, understand, and accept the meanings of self-concept, self-display, self-participation, and self-renewal (Brown, 2011; Robbins, 2015; Durkheim, 2016). In the process of interactions, the people’s meaning or value will also be transferred to the situation where the people would like to be involved in future activities, thus creating a situation where people’s generation of wellbeing continues to watch the live broadcast. See the framework in Figure 1.

## Research method

### Sample characteristics

Qualitative research methods are one of the mainstream research methods in psychology (Del Rio Carral and Tseliou, 2019; Diwanji et al., 2020; Castell et al., 2022) and have the advantage of allowing observation and communication with research subjects in a more natural setting, facilitating observation and understanding of their behaviors, attitudes, and motivations from their perspective (Knox and Burkard, 2009; Trafimow, 2014) and are well suited for this study to explore why people’s viewing of Netflix live streams can produce wellbeing in the context of COVID-19.

In qualitative research, the method of in-depth interviews is suitable for understanding complex, abstract issues. Such questions are often not clear in a few words, and only through free conversation and in-depth exploration of the topic of interest can the information to be learned to be generalized from them. And in the context of COVID-19, the question of why consumers get a sense of wellbeing from watching Netflix live is a complex and abstract one. Only through in-depth interviews can the underlying motivations, beliefs, attitudes, and feelings behind this phenomenon be revealed. Therefore, this study uses the method of in-depth interviews to explore what interactive rituals people engage in when watching live broadcasts and the reasons for and psychological mechanisms of people producing wellbeing. We recruited participants mainly through snowball sampling, asking selected respondents to identify other potential participants who shared the characteristics of interest for this study (Patton, 2014). In line with recent research (e.g., Grougiou and Pettigrew, 2011), respondents were also recruited in multiple off-line (e.g., university hall) and online (e.g., forum dedicated to mobility) channels. For this opt-in recruitment process, interested persons contacted us to participate in the study. The sampling process ceased at theoretical saturation when the information gathered became redundant, and no new information appeared in the data (Patton, 2014).

In developing our samples, we sought to maximize diversity among the respondents to achieve a holistic view of people’s generation of the wellbeing of live broadcasts while still ensuring that they shared some characteristics to facilitate comparisons of the results. The resulting sample included 56 persons, consistent with recommended sample sizes for exploratory research (McCracken, 1988). They are all loyal people who watch live broadcasts, and all of them have at least 3 years of experience in using live-broadcast e-commerce. Among them, 30 are female, and 26 are male, and their ages range from the 18s to early 40s, with a mean age of 25 years.

### Interview guide

Referring to Bonsu and Belk (2003) study, this paper adopts a step-by-step interview method: First, the moderator asks the interviewees what interactions the anchors include in their performances, interactions, and promotional product compositions during live broadcasts, and asks the interviewees to list the interaction-related rituals they noticed and to recall in detail the interactive rituals they experienced during watching the live broadcast and describe their emotions, feelings, and evaluations before, during, and after watching the live broadcasts. Second, the interviewees are asked to express their views on live broadcast and its importance, recall what their interactions with the anchor meant to them, and describe it in detail and specifically how these meanings influenced people’s generation of wellbeing. Third, the moderator continuously asks a series of questions to guide the interviewees to reflect on the reasons for these feelings and promote them to identify the influencing factors.

### Analysis and interpretation

The interviews lasted 30–95 min, with an average of 53 min. All discussions were audio-recorded and transcribed verbatim before being coded and analyzed with NVivo (Version 11). See Table 1 for basic information about the interviewees. The transcription resulted in approximately 600 single-spaced pages of text. We analyzed the data using a discovery-oriented, thematic analytic approach, that is, an iterative process of reading, assessing, and identifying emerging themes and categories that organize and describe data in detail (Braun and Clarke, 2006). The thematic analysis followed a two-step procedure: First, we coded the verbatim transcript, paragraph by paragraph, to identify relevant themes. Theoretical codes identified from prior literature (Karahanna et al., 2006) were established prior to starting the coding. Then inductive codes were added throughout the process to capture themes as they emerged from the data (Patton, 2014). At this stage, we developed a coding plan to (1) list all identified people to name the rituals, meanings, and reasons for continued viewing of interactions with anchors; (2) label and define each construct, and (3) offer typical statements to illustrate the meaning and content of each construct. The author team reviewed and discussed this plan for internal consistency, leading to some refinements to the labels and definitions. Second, we jointly developed theoretical, abstract categories for the identified constructs. We constantly compared the emerging findings with supplementary literature during the categorization procedure to integrate and extend prior knowledge (Strauss and Corbin, 1990). The findings of this study are obtained from this analytical procedure.

**TABLE 1:** Basic information of interviewees.

Record ID No.	Last name	Gender	Year of birth	Career	Interview time (minutes)
1	Ms. Liu	Female	1991	Operational researcher	36
2	Ms. Yau	Female	1993	Company secretary	47
3	Mr. Zhao	Male	1992	Legal secretary	50
4	Mr. Door	Male	1989	Business development manager	33
5	Ms. Lee	Female	1991	Project manager	50
6	Mr. Duan	Male	1993	Member of parliament	70
7	Ms. Zhao	Female	1990	Health records clerk	80
8	Mr. Song	Male	1992	Charity fundraiser	95
9	Mr. Liu	Male	1988	Farrier	30
10	Mr. Liu	Male	1992	Gamekeeper	30
11	Miss Wu	Female	1985	Rural surveyor	60
12	Mr. Dong	Male	1983	Cemetery worker	60
13	Ms. Lee	Female	1992	Dog groomer	60
14	Mr. Lee	Male	1988	Veterinary worker	50
15	Miss Ye	Female	1991	Fish farm worker	40
16	Mr. Hu	Male	1993	Landscaper	80
17	Ms. Chen	Female	1988	Tree surgeon	80
18	Ms. Jiang	Female	1985	Zookeeper	60
19	Mr. Lu	Male	1987	IT trainer	60
20	Ms. Zhang	Female	1986	Software engineer	60
21	Ms. Duan	Female	1994	Operational researcher	55
22	Mr. Qu	male	1992	Software engineer	60
23	Miss Kou	Female	1996	Veterinary worker	30
24	Ms. Xu	Female	1998	Health records clerk	90
25	Mr. Huang	Male	1992	IT trainer	70
26	Mr. Zhang	Male	1995	Cemetery worker	60
27	Mr. He	Male	1990	Business development manager	45
28	Miss Wu	Female	1994	Project manager	60
29	Ms. Ding	Female	1989	Zookeeper	50
30	Mr. Liu	Male	1994	Member of parliament	36
31	Ms. Wan	Female	1996	Charity fundraiser	48
32	Mr. Zhou	Male	1991	Legal secretary	55
33	Miss. Qian	Female	1995	Business development manager	35
34	Ms. Wang	Female	1992	Rural sureyor	75
35	Mr. Kim	Male	1997	Landscaper	65
36	Ms. Zhao	Female	1990	Health records clerk	80
37	Mr. Song	Male	1992	Charity fundraiser	95
38	Mr. Liu	Male	1988	Farrier	35
39	Mr. Lu	Male	1988	IT trainer	45
40	Miss Lee	Female	1996	Software engineer	55
41	Mr. Yu	Male	1991	Landscaper	70
42	Mr. Ping	Male	1993	Cemetery worker	66
43	Ms. Dang	Female	1993	Dog groomer	55
44	Mr. Wu	Male	1986	Veterinary worker	40
45	Miss Zhao	Female	1998	IT trainer	90
46	Mr. Hu	Male	1993	Landscaper	80
47	Ms. Cao	Female	1986	Tree surgeon	80
48	Ms. Wang	Female	1989	Zookeeper	70
49	Mr. Qian	Male	1992	IT trainer	30
50	Ms. Zhang	Female	1990	Health records clerk	55
51	Ms. Lee	Female	1991	Operational researcher	85
52	Mr. Liu	Male	1994	Legal secretary	65
53	Miss Zhu	Female	1993	Veterinary worker	60
54	Ms. Zheng	Female	1988	Operational researcher	45
55	Ms. Hao	Female	1999	Dog groomer	55

### Trustworthiness assessment

We ensured the trustworthiness and credibility of our findings by applying both data and researcher triangulation. First, we constantly compared our data with supplementary research streams (Corbin and Strauss, 2014). Second, throughout the analysis, we carefully checked the interactive rituals of the identified people watching live broadcasts, resulting in different live interaction meanings. All concepts were transferable, though some differences of magnitude arose for customers across the different adoption states. Third, for researcher triangulation, the authors discussed the coding plan and jointly categorized the findings while ensuring internal consistency and seeking agreement through discussion.

In terms of reliability, two independent judges—both familiar with qualitative research—reviewed the coding plan and then used it to code the verbatim data of 15 randomly selected interviews (27% of the sample), which exceeds the recommendation to use 10% of the sample to achieve representativeness (Lacy and Riffe, 1996). The underjudge reliability scores, calculated using the proportional reduction in loss measure for both uses’ interactive rituals of live-broadcast (0.89) and interactive meaning of different live-broadcasts (0.72), exceeded the 0.70 threshold recommended for exploratory research. Then in a second step, we asked three new independent judges to assign the themes and statements of the coding plan to the abstract categories. The underjudge reliability scores for the uses’ interactive rituals of live-broadcast (0.97) and interactive meaning of different live broadcasts (0.88) were satisfactory. Finally, we discussed the results with three experts in the service research field and the Internet celebrity ancho before presenting and discussing the findings during an academic workshop. Both the analytical process and the trustworthiness assessment provide us with the confidence that our findings capture the key people who experience and understand the interaction ritual chain of live broadcast and the meaning of interactive live broadcast.

## Findings

### Interaction ritual chains of live-broadcasts

As we introduced in previous sections, viewers in live broadcast e-commerce can use screen bullets to express their opinions, exchange views, and interact with the anchor. In those activities, many rituals have been developed and adopted. We consider interaction rituals necessary to activities in live broadcasts, which connect and link different parts of activities in a live-broadcast together. We examine the rituals-related issues in live broadcast, particularly interaction ritual chains. There are four criteria for establishing stable interaction ritual chains: (1) Virtual group aggregation emphasizes the necessity of being present in person as the starting condition of the ritual. (2) Threshold barrier, participants set boundaries for outsiders to let them know who the same audience is, so as to create a meaningful environment and spatial experience of boundary; (3) Strengthening participation conveys and knowing each other’s focus of attention in real-time, leading to heated discussions, thus producing strong resonance. (4) Emotional arousal enables participants to share emotional/cognitive experiences and connect excited, emotional states (Collins, 2014).

This study argues that the process of people who view live-broadcast e-commerce satisfies the conditions for the generation of interaction ritual chains:

#### Group aggregation

The live broadcast breaks through the limitation of physical space and creates a platform where although people cannot achieve group aggregation in person, they can share their ideas, thoughts, feelings, or emotions to achieve “virtual group aggregation” through real-time video, audio, and screen bullet technology.

In this study, the ritual of “virtual group aggregation” refers to li Jiaqi’s live broadcast (Jiaqi Li is a well know live-broadcast anchor) on Taobao Live (a well know live-broadcast platform) at exactly 8:15 p.m. on the night of the live broadcast. For fans of Li jiaqi’s live broadcast, this time point is full of a sense of rituals.

“… During the isolation of the COVID-19 at home, when I saw the live products through the push preview, I started to choose what to ‘grab’ and then set the alarm for 8:10 PM in case I missed the start of the live broadcast.” (ID1)

“…… During the isolation of COVID-19, after seeing the push preview on Weibo, I usually tap the time hyperlink directly on Weibo to automatically generate a schedule in my mobile calendar. The phone calendar will remind me when the time is up, so I won’t forget it.” (ID2)

“… At the beginning of COVID-19, I usually watch what items will be on the shelves on the broadcast day. In last week’s preview, I saw a skincare product I’ve wanted to buy, and the discount was great, and it could be a lot cheaper than buying it at the counter. I started to get nervous half an hour before the broadcast, fearing that I would miss the opportunity if my hands were too slow or if I had a sudden internet connection problem. Sometimes I even call my family members in advance to squat together to help grab items I want together, one more person, one more chance.” (ID31)

During the live broadcast, the ritual of “virtual group aggregation” provides the main scenario for interaction between the anchor and the participants. Rituals built in the online environment are no longer one-way communication of a one-to-many nature but become two-way communication with a robust interactive nature. Instead of being passive receivers, participants can actively participate in activities by considering and using the rituals involved; rituals play crucial roles in those activities. The live-broadcast channels allow participants to send real-time screen bullets to ask questions or join in discussions with the anchors; the screen bullet function of the live-broadcast room gives participants a strong sense of community, where rituals support the forming of such communities.

#### Threshold barrier

One restriction on the setting of the live-broadcast platform is that people need to have a registration account and login ID, which meets the principle of exclusion of the Outsiders (“threshold barrier”).

Setting a threshold can effectively discriminate insiders from outsiders, which helps to unite the insider interaction group. This threshold also makes fans psychologically believe that they join and enter a group with people sharing common interests and establishing a sense of belonging. After joining the group, they may uphold a more positive attitude to participate in discussions or activities.

“… into the group can directly contact the assistant to ask some goods on the shelves, it is more convenient. So, I have what I want to buy directly in the group or to ask whether there will be a special show recently.” (ID16)

For the live-broadcast operator, setting a “bar” for registered members only for a channel can prevent random visitors from mixing into the fan group to a certain extent. It can also help ensure that the group members have higher loyalty in general.

#### Strengthening participation

Having the same focus is a prerequisite and foundation for developing symbols of shared meaning and creating emotional connections. During interactive rituals, participants focus on an object and communicate and share focal information to understand each other’s practices.

“When the epidemic was severe, many shopping malls were closed. Therefore, I watched the live stream for online shopping at home. Staring at my phone, I would look at what brand of clothes the anchor was wearing today and what color lipstick was wearing. When I saw something that looked good, I would ask what product was used, and there would be others in the screen bullet begging for the same product.” (ID19)

“Sometimes I just put aside the live broadcast and do other things myself. Li Jiaqi’s voice is magical; not looking at the screen; listening to him talking, I pick up my phone to see and make an order.” (ID11)

#### Emotional arousal

Participants share their concerns and emotions during the interaction. Strong emotional resonance could be created in the process of sharing and talking. In such a state, participants can often freely voice their opinions, attitudes, and emotions genuinely.

“Due to the epidemic, certain products have experienced slow sales due to stock turnover issues. Sometimes, after waiting for a long time, I have the attitude of “xxx” (referring to the product) is finally available, or this time I must get it. After a long waiting, I will share my happy feelings in the screen bullets when the product is available on the shelves.” (ID 12)

In summary, the theory of interaction ritual chains points out that a high degree of mutual subjectivity and a solid emotional connection — through the joint coordination of the body and the nervous system evoked by the participants — are combined, making the rituals work (Collins, 2014). From this perspective, the interaction ritual of live broadcasts is a ritualized expression of the contact between the anchor and the people, and its essence is a multidimensional interactive behavior. Based on this theoretical basis and interview data obtained, this study finds that the interactive ways are divided into two categories: the interactions between the anchor and the people and the interactions between the people and the people. The screen bullet messages can be regarded as a dialogue between the anchor and the people in live-broadcast channels. In interaction rituals of live broadcasts, interaction rituals appeared during the anchor’s performance, and screen bullets were exchanged. The content of live broadcasts that viewers follow forms the focus of visual attention, which triggers shared emotions. All interactive activities, together with interaction ritual chains, strengthen the collective emotions, and this process can also become a circular interactive ritual chain. People form a common focus of attention based on live broadcasts’ content and create collective excitement during live broadcasts. This collective excitement is mainly reflected in the screen bullets. The function of the screen bullet stimulates people’s sense of interaction. The people also demonstrate a high level of enthusiasm and creativity, which attract more people to join the interactive activities, and gradually develop into a sense of identity and belonging to the group on the live-broadcast channel. When interaction rituals in live broadcasts are created and accepted by people smoothly, positive interaction results will be produced. Members’ sense of belonging will be enhanced with clear group identities and symbols. They will actively maintain the unity of this group, resulting in the establishment of the meaning of live-broadcast interactive rituals.

### The composition of the basic meaning of live-broadcast

The basic meaning of live broadcasts is the pre-factor and the fundamental cause for the development of the meaning of the interaction ritual chains, including two levels of emotional meaning and functional meaning. As far as these two basic meanings of live broadcasts are concerned, emotional meaning refers to how people will experience emotional fluctuations and generate wellbeing.

“During the epidemic, the movement was restricted due to home isolation, leading to depression. It indeed creates an emotional exchange. When helping other people better understand the product’s features, they reply to any thank you, which makes me feel responded to and satisfied. This will make me want to stay in this live room even more and feel happy.” (ID14)

“Yes, I think some anchors are very good and will answer the questions very thoughtfully. At the same time, as viewers, we can actually feel it; some anchors made a great effort. My emotional needs are definitely satisfied, and I feel happy that every time I enter the live-broadcast channel, the anchor calls my ID and welcomes me. It eased my anxiety about the epidemic, so I will continue to watch the anchor live and feel happy for the foreseeable future.” (ID17).

The content creation behaviors of people in the live-broadcast room include “emojis,” “comments,” “screen bullet” and so on, which can affect the sense of control of anchors and make people feel that the live-broadcast channel is full of an entertainment atmosphere, which is also a feature of live-broadcast. The purpose of control that people gain through cooperation and participation in cyberspace enhances their internal satisfaction (Sirlopú and Renger, 2020). At the same time, people’s behaviors in creating and distributing content can satisfy their emotional needs to some extent, and the realization of such emotional needs can further enhance satisfaction. This is a meaningful way to express emotional needs. In seeking pleasurable experiences and releasing inner feelings, people’s satisfaction is greatly enhanced, thus increasing their willingness to continue watching.

The functional meaning means that people involved in live-broadcast activities can obtain product-related information and purchase the products they expect to buy. At the same time, enjoy watching the live broadcasts or may learn helpful knowledge.

“The anchor can introduce the product well, reflect on the advantages and disadvantages, and explain the product’s source, origin, and other information in more detail. Therefore, during the COVID-19, I felt that I could learn a lot from watching the live broadcast, so I felt so happy.” (ID20)

“For example, Li Jiaqi^1^ has worked as a counter guide for L’Oreal and in the beauty product field for many years. He is familiar with various brands, and the products he recommends are authoritative. The most important thing is that he can introduce the product very clearly, and meticulously interpret the function and material used in the product. His introduction is logical and authoritative; first, it shows the product’s exterior, then the interior, etc. Therefore, when planning to shop during an outbreak, I will often watch his live show to buy the products he recommends. To make myself less boring and not particularly empty mentally.” (ID21)

Berchicci and Tucci (2010) defined people’s information acquisition needs as the people’s willingness to want to learn and collect various types of online information content. Moreover, people’s need for information content can stimulate them to act accordingly. People’s need to know and acquire information is the driving force for their consumption of live-broadcast channels. It is also essential for their immersion in online social media (Diwanji et al., 2020). Based on the above, the following propositions are made:

P1: The basic meaning of live-broadcast interaction positively affects people’s generation of wellbeing.
P1a: The emotional meaning of live broadcast positively affects people’s generation of wellbeing.
P1b: The functional meaning of live broadcast positively affects people’s generation of wellbeing.

### The composition of interaction ritual chains

Rituals can be seen as an aggregation of various symbols, and their essence lies in revealing the meaning of value, thus exerting the purpose confined to the core meaning of a brand, for example (Collins, 2014). Therefore, individuals could understand their values through the interaction rituals of live broadcasts and stimulate emotional resonance and identification. Four types of self-related value meanings have been recognized in this study in live broadcasts: self-concept, self-display, self-participation, and self-renewal. First, the value of self-concept is that consumers form an understanding of self-identity through the interaction rituals of live broadcasts. Live-broadcast interaction rituals often repeat their processes and symbols in the execution process. The purpose is to enhance the atmosphere of the rituals and the authority of the rituals (Rook, 1985). In the ritual atmosphere, people can improve their understanding of their identity and concepts.

“I like to watch my favorite celebrities’ live broadcasts, and they will market some local specialties through live broadcasts. On the one hand, it’s to support their favorite artists, and on the other hand, it’s to try different local specialties. It also gives me a great sense of well-being.” (ID22)

“Due to the epidemic, cooking your own food at home is more frequent than usual. As a result, it has become a major challenge to make different specialties, which is why I watch live broadcasts, especially of gourmet specialties. The reason I like to watch food specialties is that I think I can buy cheap and delicious special food from all over the world by watching specialties live broadcasts, and I can also see the local customs and local people. It is also a blessing to recall the stories of home.” (ID30)

“I like to watch the live broadcast of my hometown’s specialties, and I like the life- broadcast mainly because of the hometown complex, and buying the specialties of my hometown will remind me of my days in my hometown. Hometown is synonymous with happiness.” (ID24)

Second, self-expression refers to the people’s display and express themselves through the process of the interaction rituals of live broadcasts. The rituals have the characteristics of symbolic appearances and performance features. As a result, the artistic and dramatic surroundings present an excellent environment that everyone yearns for and constitute a desirable platform for individuals to express themselves (Robbins, 2015).

“During the buying process, I would share what I thought of with the anchors and other people through the bullet screen and ask for their approval.” (ID26)

“When watching live during an outbreak, I will respond positively and interact with them to discuss topics related to the purchase of the same products together, and respond positively to questions and suggestions made by the other party.” (ID33)

Third, self-participation is when people perceive a sense of participation through the process of the interaction rituals of live broadcasts. Participation is a manifestation of people integrating into society and being accepted by a community. The interaction rituals of live broadcasts divide individuals into accepted insiders and not taken outliers in interactions (Brown, 2011). As a result, the interaction ritual chains of live broadcasts can bring consumers a sense of meaning and value for participation.

“During the purchase process, if it is a product I have already purchased and I am delighted with it, I will proactively communicate with other people who are interested in purchasing it.” (ID28)

“Yes, I will; when I meet someone like me, I will interact with them.” (ID29)

Fourth, self-renewal is the experience of renewal, change, and improving people themself through the interaction rituals of live broadcasts. The original function of ways is to “transition” (Durkheim, 1965). Many ceremonies, such as adult rituals, wedding rituals, graduation ceremony rituals, etc., represent the transition of people from one state to another. Previous studies have found that rituals can help individuals accept new roles and live quickly and smoothly (Norton and Gino, 2014). Therefore, this study proposes that the interaction rituals of live broadcasts can generate the meaning of self-renewing.

“The epidemic has limited many offline social activities, I participated in the interaction in the live broadcast room and met many like-minded friends; through the interaction with the anchor and fans, I learned much knowledge; being with everyone every day allows me to have a happy time, and the troubles in life are wiped out.” (ID35)

The fusion effect of the meaning of the interaction ritual chains of live broadcasts on the basic meaning of live broadcasts and people’s generation of wellbeing coincides with the essence of the meaning transfer model. The meaning transfer model explains the symbolic flow and changes of individuals, objects, and related rituals in the consumption process from the perspective of social construction (McCracken, 1989).

“Every day, I would wait for the extensive wine god’s^2^ live-broadcast on time, and I would interact with him inside the live-broadcast room, say hello to him, and he would read my id. Now, I have developed a habit. During the COVID-19, It was very satisfying to watch his live broadcast. Even though I haven’t played games for a long time, I just love watching him play and listening to his commentary; his live broadcast is my source of happiness in the evening.”(ID30)

“I watch his live broadcast During the COVID-19, and I love the feeling of people talking together on his live broadcast; I have also joined his fan support group and met many friends. I will post my opinion on the screen through the bullet screen, and the anchor and everyone will discuss it together. I am thrilled to get everyone’s approval so that I will attend on time every day.” (ID31)

“I listen to Little Seven^3^ sing every day; she sings very well and is very funny and exciting. I also give her gifts from time to time, and she will chant my name, which makes me feel very valuable. Moreover, everyone will send me envious emojis, which can satisfy my vanity, make me feel more accomplished in the virtual world, escape from the worries of reality, and allow me to meet my true self. I am so engrossed in this environment that I cannot help myself; even at night, when I am working late, I will watch live broadcasts while I am working.” (ID14)

“I watch every live broadcast of Li Jiaqi, although I don’t always buy something. I think I can learn a lot about the products by watching his live show. He will explain the difference between the products and the people for whom they are suitable, so even if I don’t need to buy anything, I will still insist on watching his live show every day, and his live broadcast has become part of my life.” (ID33)

Through the interactionsin the context of live broadcasts, people realize the meaning of self-participation, self-display, self-concept, and self-renewal. People concentrate on and engage with interactive activities, which would produce a sense of pleasure. People regard such activities more as a kind of reliance and sustenance, hoping to break away from reality into the live-broadcast platform to reach the narrative world and then return to reality with the emotion and attitude of the narrative world. When the feedback of the interaction ritual chains affects the operation of the subsequent interaction ritual chains, it creates a long-term feedback effect; the platform will further affect the people’s experience and strengthen the people’s dependence on the platform so that the people watch this anchor on this platform again. This conclusion is also consistent with the research conclusions on mobile network services; when using mobile network services, people satisfied with the application or service experience are more likely to generate a sense of wellbeing (Xu et al., 2021).

Similarly, in social networks, when people have satisfactory experiences where their social needs and self-fulfillment needs are met, their willingness to people’s generation of wellbeing (Chang et al., 2015). Therefore, in each interaction ritual of live broadcasts, participants gather through the focus of mutual attention and obtain a sense of personal satisfaction. This emotional energy and the extension of its symbolic meaning are the fuel to push participants to join the next round of interaction ritual chains. As a result, the people generate a sense of wellbeing.

Based on this, the following propositions are made:

P2: The meaning of self-concept plays a role of mediation in the basic meaning of live interaction for people’s generation of wellbeing.
P3: The meaning of self-display plays a role of mediation in the basic meaning of live interaction for people’s generation of wellbeing.
P4: The meaning of self-participation plays a role of mediation in the basic meaning of live interaction for people’s generation of wellbeing.
P5: The meaning of self-renewal plays a role in mediation in the basic meaning of live interaction for people’s generation of wellbeing.

## Discussion

### Theoretical contribution

First, from the perspective of the interactive ritual chain proposed in this research, this study examines people who continue to watch the live broadcast behavior path, which expands and improves the existing research results of live broadcast people behavior. Existing research on live broadcasting mainly focuses on the characteristics of the anchor’s information source (Jiang et al., 2022), the anchor’s attractiveness (Peng et al., 2020), the social motivation (Hilvert-Bruce et al., 2018), and the quasi-social from the perspective of interaction and other issues. There are still few studies on the impact of live broadcasts on the people’s continuous adoption behavior and internal psychological mechanisms. Based on the interactive ritual chain theory and the meaning transfer model, it is found that people who watch live-broadcast and interact with anchors will form a live interactive ritual chain, which generates emotional energy, thus making people understand and experience the meaning of live interaction. Under the meaning transfer model, people will develop people’s generation of wellbeing. This research demonstrates and verifies that the ritualized behavior in live broadcast interaction fully integrates people’s cognition and emotion of live broadcast and is distinguished from religious rituals and brand rituals (Ahler and Tamney, 1964). The results of this study form a helpful supplement to the existing results in ritual research.

Secondly, this research enriches and expands the meaning transfer model. Previous studies on meaning transfer modeling find that rituals originated from the flow of symbolic meanings. Among them, cultural symbols are the foundation. At the same time, advertising and fashion are systems of reflection, which are then reflected in consumer behavior, and then affect consumers’ psychology through consumer behavior (McCracken, 1989). This study suggested that the formation of the interaction ritual chains of live broadcast is the flow between the basic meaning of live broadcast and the meaning of the interaction ritual chains, which confirms the flow of symbolic meaning. This study found that the meanings of self-participation, self-display, self-concept, and self-renewal play a role in mediation in the basic meaning of live interaction to enhance people’s generation of wellbeing. These findings will benefit subsequent studies of interactive ritual chains.

### Managerial implications

The outcomes of this study provide insightful information to form people’s wellbeing habits from a management perspective.

This study finds that people who watch live-broadcast and interact with anchors will form a chain of live interaction rituals and generate emotional energy, thus making people develop a sense of belongingness and togetherness. These leads people to continually be involved in activities and watch these live broadcasts.

One is to hold a ritualized live-broadcast management concept for anchors. So, anchors could promote the use of interaction rituals in their performance, constantly improving their performance qualities and providing high-quality content to maintain the show’s attractiveness. Creating a good communication atmosphere in live broadcasts and encouraging people to develop and form interaction ritual chains is essential. Anchors need to pay attention to activities involving interactions and invite viewers to contribute and be involved. The anchor could integrate elements of emotions and functions in content planning. For live-broadcast platform managers, attention must be paid to creating an ecological system to increase the platform’s competitiveness, which is essential to attracting high-quality anchors.

### Limitations and research directions

This study has a few limitations. First is the end of segmentation of the research object. This study does not classify the research object into different categories. However, in future studies, people may be grouped based on their needs; second, this study examined it from the angles of emotion and function; furthermore, based on the results of interaction ritual chains formed, other tips may need to be considered. Third, in this study, a theoretical model of the meaning of live consumer interaction has been developed through qualitative analysis. Future studies could be conducted using questionnaires to collect data to confirm the propositions proposed, the themes, and their relationships identified, and further construct a model to testify and verify the influencing, mediating, or moderating effects of various factors and variables.

Fourth, with the progressive development of technology, the real-time images presented by live broadcasts can make people face the screen to join the ritual, but can it completely replace the effect of offline physical contact with the ritual? Will technology development, such as AR and VR technologies, produce a sense of immersion and live like reality? Will it become a science-fictional future that completely replaces offline rituals and overturns all current modes of social interaction? Those are the questions that may be considered in future studies.

## Data availability statement

The raw data supporting the conclusions of this article will be made available by the authors, without undue reservation.

## Author contributions

LM was responsible for the construction and later revision of this manuscript. YZ was responsible for the later revision of this manuscript. YJ was responsible for theoretical literature review, article writing, and adjusting. YB was responsible for the adjusting. JL was responsible for article language touch-up and data collection, sorting out the data, and related auxiliary work. All authors contributed to the article and approved the submitted version.

## Footnotes

1 Li Jiaqi is an e-commerce anchor, beauty expert nicknamed “Lipstick Top,” and a partner of Beauty Wrist (Shanghai) Network Technology Co.

2 Wine God: former Dota professional player, played for AVNC, LOST, ONCE, EHOME, FTD; current gaming video commentary.

3 Xiao Aqi: well-known female singer, YY Live Gold Artist.
